# Do Communities Really “Direct” in Community-Directed Interventions? A Qualitative Assessment of Beneficiaries’ Perceptions at 20 Years of Community Directed Treatment with Ivermectin in Cameroon

**DOI:** 10.3390/tropicalmed4030105

**Published:** 2019-07-15

**Authors:** Fanny Nadia Dissak-Delon, Guy-Roger Kamga, Perrine Claire Humblet, Annie Robert, Jacob Souopgui, Joseph Kamgno, Stephen Mbigha Ghogomu, Isabelle Godin

**Affiliations:** 1 Ministry of Public Health, N°8, Rue 3038 quartier du Lac, Yaoundé, Cameroon; 2 Ecole de Santé Publique - Campus Erasme, Université Libre de Bruxelles, Route de Lennik 808 CP591, 1070 Brussels, Belgium; 3 Molecular and Cell Biology Laboratory, Department of Biochemistry and Molecular Biology, University of Buea, P.O. Box 63 Buea, Cameroon; 4 Institut de Recherche Expérimentale et Clinique, Université Catholique de Louvain, Clos Chapelle-aux-champs 30 bte B1.30.13, 1200 Brussels, Belgium; 5 Institute of Molecular Biology and Medicine, Université Libre de Bruxelles, Rue des professeurs Jeener et Brachet 12, 6041 Charleroi (Gosselies), Belgium; 6 Centre for Research on Filariasis and other Tropical Diseases, P.O. Box 5797, Yaoundé, Cameroon; 7 Faculty of Medicine and Biomedical Sciences, University of Yaoundé 1, P.O. Box 1364, Yaoundé, Cameroon

**Keywords:** community participation, community directed interventions, qualitative survey

## Abstract

Recent studies in Cameroon after 20 years of implementation of the Community Directed Treatment with ivermectin (CDTI) strategy, revealed mixed results as regards community ownership. This brings into question the feasibility of Community Directed Interventions (CDI) in the country. We carried out qualitative surveys in 3 health districts of Cameroon, consisting of 11 individual interviews and 10 Focus Group Discussions (FGDs) with specific community members. The main topic discussed during individual interviews and FGDs was about community participation in health. We found an implementation gap in CDTI between the process theory in the 3 health districts. Despite this gap, community eagerness for health information and massive personal and financial adhesion to interventions that were perceived important, were indicators of CDI feasibility. The concept of CDI is culturally feasible in rural and semi-urban settlements, but many challenges hinder its actual implementation. In the view of community participation as a process rather than an intervention, these challenges include real dialogue with communities as partners, dialogue and advocacy with operational level health staff, and macroeconomic and political reforms in health, finance and other associated sectors.

## 1. Introduction

1978 was a crucial date year for Public Health, marked by the well-known Alma Ata Declaration, in which health was reaffirmed as a human right, and by, Primary Health Care (PHC) being recognized as essential for achieving the goals of “health for all by the year 2000” [1]. However, during the four decades that followed 1978, the promises were not reached, and despite the efforts of PHC rebirth in 2008 [2,3,4], the 40th anniversary of Alma Ata was marked by slow progresses in PHC, namely in low and middle income countries [5]. In October 2018, the international community gathered once again in Kazakhstan to reaffirm the principles of PHC and universal health coverage in what was called the Declaration of Astana [6]. Community participation (CP) is the basic principle that supports and is at the origin of PHC [2]. However, many authors also identified CP, as one of the weakest links in PCH [2,4,5].

The first challenge of community participation lies in the lack of consensual definitions of the terms “community” and “community participation”, especially between academicians and health policy makers [3,7]. In addition, CP has also faced multiple ways of implementation over the years, as a result of the bipolarization of the PHC paradigm into two opposing approaches: The vertical or focused approach versus the comprehensive or horizontal approach [2,4,5]. In fact, during the 3 first decades following Alma Ata, the dominance of the selective understanding of the PHC principles led to the fragmentation of many health systems, namely in Sub-Saharan African countries, into vertical health programs that focused on individual communicable diseases control [3,5,8]. Consequently, CP was implemented through multiple exclusive community based interventions in the framework of those health programs [2,3]. Within that context, the term community finally referred to a population that live in the same circumscribed geographic area [3]. The last ten years, dedicated to PHC engagements renewal, have been marked by a progressive switch to the comprehensive approach with better community empowerment [4,5]. One example of the effect of this switch in CP in Sub-Saharan Africa, is the experience of the African Program for Onchocerciasis (APOC) with the Community Directed Treatment with Ivermectin (CDTI).

The CDTI was implemented by APOC, as a response to the difficulty of ensuring adequate therapeutic coverage that was experienced in the field, despite the donation of ivermectin by the manufacturer [9,10]. Prior to CDTI, the Community Based Ivermectin Treatment (CBIT) was used for mass drug administration. However, CBIT had several limitations, including costliness and ineffectiveness in terms of therapeutic coverage and population ownership [10]. While the CBIT relied on local health staff, the CDTI is a population-centered strategy that encourages the beneficiary populations to take entire responsibility for their treatment [11,12]. Moreover, contrary to other interventions involving CP, the CDTI process has the advantage of a well-articulated system in which the roles of the different actors are clearly defined [12], and has shown better results in terms of treatment coverage and community health empowerment [13,14,15,16]. By the year 2005, due to the success of CDTI, the concept of Community Directed Interventions (CDI) was born. The idea here was to achieve integrated comprehensive community interventions with real ownership by the populations [17]. Many authors demonstrated the feasibility, success and added value of implementing other health interventions by using the CDI approach. Examples of such experiences include other neglected tropical diseases, vitamin A supply, malaria control (delivery of Insecticide Treated Nets and home-made management) and directly observed treatment of tuberculosis by using the CDI approach [18,19,20,21].

As member of the former APOC, Cameroon has been implementing the CDTI for the past two decades and was involved in many of the multi country studies that demonstrated the success of CDTI. Like many other countries before, Cameroon decided in 2016 to adopt the CDI approach in several core health interventions such as HIV/AIDS, Tuberculosis, Malaria, and Maternal, New-born, Child and Adolescent Health [22]. However, the analysis of these multi-country studies showed that from the beginning of the process, Cameroon had some difficulties implementing CDTI and had lower or mixed results in terms of community ownership, community distributors’ performances, and treatment coverage [9,15,23,24]. Recent studies conducted in the country, aimed at assessing the adherence to ivermectin, also showed some evidence of poor population ownership of the process [25,26,27,28]. This brings into question the transferability of the successes of CDTI in all contexts, and, more importantly, the feasibility of scaling up to CDI in the country. 

To address this question, it is critical to first have a state of play of the CDTI process in the country. We therefore conducted the present qualitative survey, which aimed to assess beneficiaries’ perceptions of their implication in the CDTI.

## 2. Materials and Methods

### 2.1. Settings

**Figure 1:**
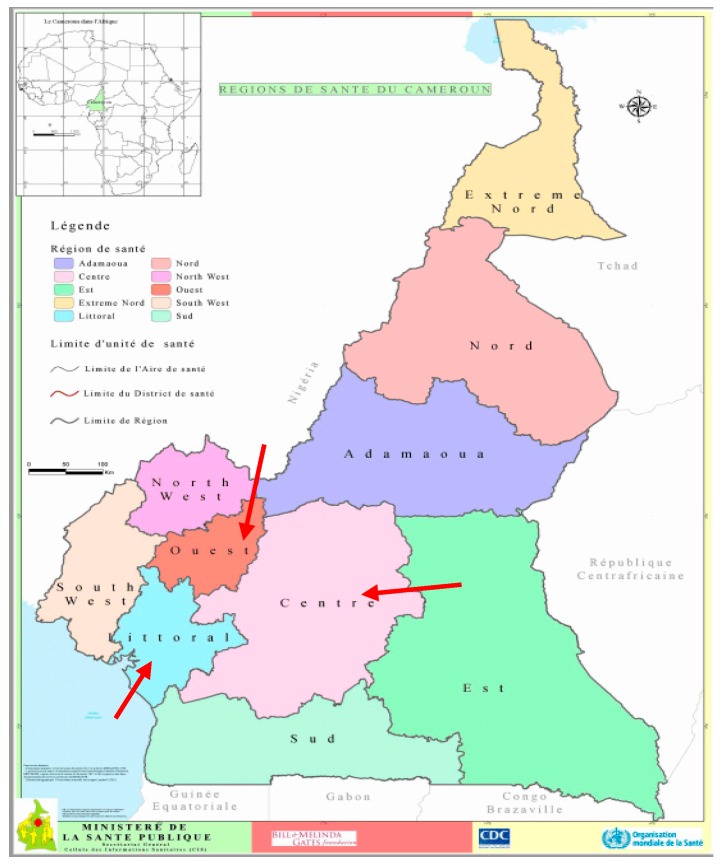
Map of Cameroon showing the study areas: The Regions of the West, the Centre and the Littoral. (Source: 2016 © Ministry of Public Health; https://www.dhis-minsante-cm.org/portal/. Administrative Research Authorization N° 631-1315).

The present study follows a first quantitative survey, which aimed to assess the determinants of beneficiaries’ adherence to ivermectin in 3 Health Districts (HDs) in the West, the Center and the Littoral Regions in Cameroon (see Figure 1 below). Those HDs were selected because of the persistence of onchocerciasis transmission [25]. In that study, we found that the beneficiaries had poor knowledge of onchocerciasis and the CDTI program [25]. Moreover, they had a mixed perception of the CDTI process in their context. We therefore devised this study to understand the communities’ perception of the role that is really given to them in the CDTI program. More specifically, we aimed to: (i) Describe the CDTI process according to the beneficiaries’ views in comparison with what was theorized by APOC; (ii) understand the beneficiaries’ expectations concerning involvement in CDTI; (iii) understand the facilitators of, and barriers to community participation in the districts of our study.

### 2.2. Theoretical Framework of Community Participation in the CDTI/CDI Process

Like the definition of the term itself, the assessment of community participation has been widely discussed by scientists. Numerous scholars propose different models of assessing or scaling community participation. In the specific context of community participation in PHC, Rifkin et al. in 1988 proposed a framework of five factors, including needs assessment, management, leadership organization and resource mobilization [29]. For each factor these authors described a continuum of participation levels according to which the examined community can be defined [29]. In 2010, Draper et al. proposed a modified version of this framework that emphasizes ownership and women’s involvement, that can be summarized in a “spidergram” [30]. In 2016, Lippman et al. developed a seven-dimensional scale to measure community mobilization. The seven dimensions proposed by these authors comprise social control, social cohesion, shared concerns, critical consciousness, leadership, organizations and network, and collective actions [31]. The above-cited authors are not the only ones to have studied and proposed measurement tools for community participation. However, as pointed out by George et al, the common point of most of the authors is that they all end up evaluating “*the level of control or power that communities command in an initiative*” [7]. According to this ownership paradigm, a “good level” of community participation is when the community has the full control of the decisions that are taken in implementing the concerned health intervention(s).

The authors of the CDTI concept relied on another community participation scale proposed by Marsden and Oakley in 1990. Nevertheless, this scale has the same premise of community ownership as the ideal form of participation [12]. The CDTI is more than a simple tool for community participation: it proposes a clearly described model of application of the most advanced form of CP, in which community members are empowered “*to make major decisions and direct the distribution of ivermectin*” [12]. 

Analyses of World Health Organization (WHO) literature [12,32] allowed us to understand that the CDTI process can be divided into a pre-community phase and community phase, as summarized in Table 1. 

The pre-community phase consists of advocacy and planning meetings at National level with Non-Governmental Development Organizations (NGDOs). This first phase ends with the training of health staff at District level and at the first line health facility. After the pre-community phase comes the community phase which is the topic of our study.

The community phase “*begins when the health worker visits the chief of an affected community*”, and is divided into 3 main steps [32]:

(1) Selection and training of volunteers: During the meeting with the community leader, the health staff explains the concept of CDTI. After that, in general meetings with all the inhabitants, the community: Decides whether or not to adopt the CDTI; decides on the schedule and the process; decides on the selection and remuneration of Community Directed Distributors (CDDs). Following these decisions, the community informs the health worker, who performs the training of the CDDs selected by the community. The first step ends with the census of the community by the CDDs, who then order ivermectin accordingly from the health worker;
(2) Ivermectin collection and distribution: At the date decided by the entire community, the selected and trained CDDs distribute ivermectin. They are also responsible for monitoring eventual side effects. Minor ones are treated by the CDDs and in case of severe side effects they refer to the nearest health facility. At the end of the treatment the CDDs send their reports to the local health staff. They also report to the entire community and the community adjusts its resolutions for the next session. At this stage the role of the local health staff is to monitor the treatment records during visits to communities;
(3) Repeating treatment each year: CDD selection and (re) training is done every year (or every two years).

The aim of this study was not to evaluate community participation in general, but within the framework of CDTI. Consequently, we used the 3 steps of the community phase as lenses for our evaluation.

### 2.3. Study Design and Participant Selection

We chose the qualitative approach to better explore communities’ experiences of CDTI. We used individual interviews and focus group discussions as research methods in order to give respondents, the opportunity to answers in a critical manner, with regards to their own context and experiences.

The field work took place in July, August and December 2016, with one month per HD. We were guided in the field by facilitators who were provided by the respective District Medical Officers. In order to ensure the variability of respondents in the HDs, we chose to work in two different communities located in two different health areas. In each HD, we took the list of the health areas and gave a number to each of them. Then we used the “random” function on the Microsoft Excel software to obtain the 2 health areas per district. The same procedure was used to select the communities of our survey (1 community per health area), based on the list of the communities of each selected health area. In all the 6 communities of our study, we conducted individual interviews and Focus Group Discussions (FGDs) (Supplementary Materials). 

We interviewed the traditional leaders of the different selected communities individually. In one of the health areas we were advised to also meet the traditional head of the canton (his authority covers all the communities of the health area). Individual interviews were also done with community members who refused to take ivermectin (CDTI-averse) during the last campaign prior to our study, in order to gather the opinion of those who openly refuse to take part to the CDTI. They were identified by the facilitator, with the help of the CDDs of the community. We stopped the recruitment of CDTI-averse when we reached saturation. We conducted FGDs with community members who agreed to participate in our study. FGD group composition was done conveniently with the help of the facilitators, who looked for volunteers 2 days prior to the meetings and made appointments with them. 

### 2.4. Data Collection

Interviews were done mainly in the homes of the participants, while FGDs took place in public places such as primary schools, village social halls, or other places selected by the participants. In order to facilitate discussion within the groups, we separated the Youth (16 to 25 years old) from the elders (35 years old and above) in each focus group. The language used in all the interviews and FGDs was French, which is the predominant official language in all the 3 regions of our fieldwork.

Individual interviews lasted 30 to 51 min and FGDs about 45 min. The main themes discussed in individual interviews were: The ways in which people usually prevent/treat diseases in their context (for community leaders there was a probe about their role in disease control in their communities); the description of onchocerciasis control activities in general in the communities; the roles of communities in onchocerciasis control and their expectations for that role. Community leaders also discussed their relationship with the other stakeholders, while the ivermectin-averse discussed their reluctance and how that related to participation in onchocerciasis control activities. Although our aim was onchocerciasis control, our respondents were free to give examples of their statements in other community-based activities that came to mind. FGDs were more focused on the description of CDTI (ivermectin distribution programme) process in the communities, participants’ role in the process, and their expectations for community participation. 

### 2.5. Data Analysis

All the interviews and FGDs were audio taped, allowing iterative analysis as fieldwork was ongoing. Findings from the preliminary analysis lead to the adjustment of probes within the initial broad topic, for subsequent interviews or FGDs.

A research assistant transcribed the data audio tapes and one author checked these transcriptions’ trustworthiness. Data analysis was done using a general inductive approach, explained by Thomas as a systematic procedure for analyzing qualitative data with regards to the study objectives [33]. Using this approach, we followed rigorous steps which included: Raw data organization, repeated thorough reading of all the transcripts, identification of the first emerging categories, and merging/refining of those categories [33]. In the case of FGDs, special attention was also paid to identifying the group interactions and possible individual opinions [34]. Three of the authors separately did raw verbatim analysis and consensual conclusions were discussed in group.

### 2.6. Ethical Considerations

Prior to fieldwork, ethical approvals were obtained from both the National Ethics Committee for Human Health Research (N°2015/01/543/CE/CNRESH/SP) in Cameroon, and the Institutional Review Board of the Université Catholique de Louvain, Brussels campus in Belgium. Additionally, the Cameroon Ministry of Public Health granted an Administrative Research Authorization (N°631–1315). We also met with the Permanent Secretary of the National Onchocerciasis Control Program, the Regional Delegates of Public Health for the West, Centre and Littoral Regions, and the District Medical Officers of the selected Health Districts, to obtain their verbal or written approvals. 

All the participants were informed about the study objectives and the audio taping of interviews/FGD before the beginning and agreed with the procedure. The following precautions were taken in order to ensure our participants anonymity:

- Informed consent was verbally obtained;
- Data were safely stored in a computer with password-coded access;
- The names of respondents and of their origins were coded in the final manuscript into “villages”, so we had village 1 through village 6.

## 3. Results

We did a total of 11 individual interviews, with 1 canton leader, 6 community leaders and 4 “CDTI-averse”. All the traditional leaders were men, while among the CDTI-averse we had equal numbers of men and women (see Table 2). 

The 10 FGDs, composed with almost equal male/female parity, were made up of a minimum 5 and a maximum 14 participants (see Table 3). We observed equal participation of men and women inside the groups during the exchanges.

Analysis of our data gave us a description of the CDTI process at a community level, according to beneficiaries’ experiences. We also understood their perceived roles in CDTI, their expectations, and finally, some elements that can influence community participation as regards the value of ownership advocated by the CDTI theory.

### 3.1. CDTI Process and Roles Distribution According to Communities

#### 3.1.1. Selection and Training of Volunteers

A large majority of our respondents had the perception that the CDTI process was limited to the day of treatment. According to them, the process starts when the CDD enters a household for measurement and treatment and ends at his departure. 

Respondents explained how the Chiefs of Health Area (CHAs) did most of the CDD enrolment, using multiple methods for selection. Usually, the CHAs relied on heads of communities, on local associations or on health center personnel from the village, to help them with the selection of a few volunteers. Those volunteers then played the role of resource person and proposed their relatives in case of a need for renewal or of a membership increase. As explained by this participant:

“*[to be a CDD] … it’s a matter of relationship, meaning that you already have your person who is in front, who is perhaps in the health, who is in a health center where the information about vaccination campaign is released. That person is asked if he knows available people to perform vaccination or to distribute Mectizan; it is now up to him to take his relatives.*”

(Female participant, FGD Youth, Village 1)

It is noteworthy that this progressive recruitment gave some members of the community an impression of discrimination and withholding of information. For instance, during the FGDs, participants used the terms “relationship” or “succession” to describe the CDDs enrolment process.

Furthermore, the decision to revoke a CDD was also the CHA’s responsibility, as illustrated by this participant’s statements:

“*This is where [the CHA’s name] said: ‘I take you off, I’ll now work with Youth. This is when he recruits the two young girls who presently share Mectizan…*”.

*(Female participant, FGD Youth, Village 3)*

The training of CDDs was done by the CHAs, at a date and in a place that they decided and communicated to the selected CDDs.

#### 3.1.2. Ivermectin Collection and Distribution

Our respondents did not clearly discuss ivermectin supply, but it was implicit that ivermectin offered by the government was brought to the communities via the CHA. More and more often, the census and the treatment took place at the same time. Only in one of the 6 health areas of our study did participants at an FGD report a census prior to the distributions. The community was not involved in the decision of the distribution strategy, whether door-to-door or in fixed distribution posts.

#### 3.1.3. Repeating Treatment Each Year

All the community leaders and most of the community members acknowledged that CDDs were trained/recycled each year prior to the ivermectin distribution campaign. However, none of our respondents had ever heard about auto-monitoring meetings at the end of each campaign. 

A comparative summary of the CDTI process between what is expected by WHO/APOC and what we found on the field is done in Table 4. It shows that most of the activities involving communities’ responsibilities were either done by the Chief of Health Area or not done, except for the report to the health system, which was done by the CDDs instead of the entire community.

Since our participants didn’t know the CDTI process very well, they didn’t spontaneously mention the monitoring of the distribution process and we didn’t specifically ask about it. However, we had specific probes to assess auto monitoring, especially during individual interviews.

### 3.2. Perceived Roles of Communities and Their Expectations for CDTI

#### 3.2.1. Passive Role of the Communities in the CDTI

In general, the community leaders that we met complained about a lack of collaboration with local health authorities. They reported to have little or no interaction with District Officials, and in most cases, they had patient-healer relationships with the CHAs (usually the heads of primary health care facilities). In the specific case of CDTI, the community leaders said that their role was to inform the populations about ivermectin distribution dates, in case they had previously been informed by the CHAs. At times, community leaders also helped the CHAs choose volunteers. However, in that case, the choice of those volunteers was not done after a general community meeting, but rather according to their personal appreciation of villagers.

“*By the time we had to set a health committee, the chiefs were asked to choose in their villages people of good character. Well, especially since it’s a volunteer job, someone should not go there ask for a salary*”

(Community Leader, Individual Interview, Village 2)

None of the villagers that we met had ever attended or heard about general assemblies organized in their communities to decide on CDTI organization. Communities clearly perceived their role in the CDTI process as a passive one:

“*We are only like the spectators*”.

(Female Participant, FGD Youth, Village 1)

From their experience, the CHAs made the decisions around the planning of ivermectin distribution campaigns and spread the information through local radios, churches or local associations. 

#### 3.2.2. Obtaining Communities’ Expectations from CDTI Was Difficult

The expectation of community leaders and communities in general was to have a health center in their own villages. Understanding participants’ expectations concerning preventive health was difficult, because people generally had an attitude of being recipients in relation to a government that provides everything and is supposed to initiate all health initiatives at the local level:

“*For the prevention …, the tragedy here in our village, and we reproach it to the health service, I would even say the departmental health service: we have no campaigns. We do not have prevention campaigns, so I would say in one word that we are not assisted (…), as concerns screenings, as concerns advices, as concerns encouragement.*”

(Community Leader, Individual Interview, Village 2)

During our conversations we understood that people’s recipient attitude in preventive health was because they thought that they had poor knowledge of health, and therefore were not skilled enough to express relevant needs in that sector. Along the same vein, in the villages without a health center, some people associated the lack of initiative with the absence of a nurse in their village.

“*We will sit on what basis? Yet if we had a health hut in this village here, we would understand that health is important since the nurses are there.*”

(Male Participant, FGD Elders, Village 3)

Finally, in the specific framework of CDTI, the main community members’ expectations concerned the access to information about the disease and the organization of CDTI. Community leaders on the other hand expressed the need to be approached by the local health authorities for better collaboration

### 3.3. Factors That Can Influence Community Participation

#### 3.3.1. Local Organization of CDTI and Information Sharing

Although most of our respondents declared they were not informed about CDTI process, community leaders and FGD participants who did have the information about volunteers’ registration complained that they did not received that information sufficiently in advance:

“*So, the information was not well relayed, it is also necessary. We must be informed before, so that we can properly prepare and organize ourselves*”

(Female Participant, FGD Youth, Village 1)

Time was an important factor in local organization. Indeed, our participants believed that being a CDD was commendable and most regretted the lack of time for this activity. 

Access to correct information about the CDTI was also crucial in local organization, particularly regarding CDD remuneration. Most of the encountered persons believed that CDD was a paid job. This idea was indirectly linked to the fact that they were “recruited” by the CHAs. The notion of remunerated CDDs was also in part due to some of the arguments the CHAs had used to convince villagers to be enrolled. For example, during a FGD, one of the participants narrated the circumstances that led to her husband becoming a CDD:

“*The chief of the [health] area told my husband that: ‘well, I see what you’re doing on the road there, it’s a waste time, (…) I must take you too, also benefit from this side’*”

(Female Participant, FGD Elders, Village 5)

This ignorance of the voluntary nature of the CDD position could be a source of misunderstanding and frustration for CDDs when at the end of the distribution they received no salary.

#### 3.3.2. Rapidly Growing Economic Background

During the FGDs with youth, we clearly understood their need to contribute to the growth of their communities. Some of them are part of spontaneous youth associations, with precise objectives:

“*You know that Madam, that youth association, what really concerns us is, really living in society, and growing up as well*”.

(Male Participant, FGD Youth, Village 6)

However, the scarcity of young CDDs illustrates how this potential taskforce is largely unengaged in the communities. This limited involvement of youth is a consequence of an economic background that makes people, especially youth, less and less interested in volunteering. As a matter of fact, in all the FGDs with elders, participants unanimously complained about young people’s lack of interest in non-profit activities. 

During the FGDs with youth, when we confirmed that CDD was a volunteer job, they acknowledged that in this case it would be difficult to have young CDDs, and confirmed the crucial role of the economic context in their reluctance:

“[F:] *Life in the village is difficult, is difficult!* [M:] *If you don’t work, you have nothing*”

(Female [F] and Male [M] Participants, FGD Youth, Village 3)

Nevertheless, we note that during the discussions some of the youth specified that it was possible for them to do part-time volunteering for their communities.

#### 3.3.3. Community Involvement in Existing Activities: Examples of Sanitation Campaigns and of Security Committees

During our fieldwork we noted community-based and even community-stimulated activities that had massive villager involvement. Two examples that were common to the 3 HDs of our study were sanitation programs and security committees. The analysis of our data showed that the common point between these two activities was the fact that they corresponded to what the inhabitants perceived as important at that time.

The “cleanliness day”, is a top-down activity driven by municipalities in which one day of the week is chosen by the municipality and dedicated to sanitation. During that day, traders must open 2 h later than usual, and everyone cleans the streets, the water points, as well as the public buildings and squares of the village. Although the activity is compulsory, during individual interviews all the community leaders noted the villagers’ enthusiasm to participate in the activity, especially in comparison with other health related programs. Community leaders explained this greater adherence to sanitation activities as a result of the prevalence of waterborne diseases and malaria in their milieu, and the general knowledge that the prevention of those diseases requires environmental sanitation. This was confirmed by the participants to our FGDs, as summarized by this statement:

“*Health starts with cleanliness*”.

(Male Participant, FGD Youth, Village 6)

Perception of stakes can lead people to unite against a common problem even beyond health. This is the case with the “self-defense committees” that people spontaneously set up in the case of an upsurge in thefts and/or assaults in their community. Most of these committees are led by young men who guard the neighborhood at night, and at times other security measures are taken, depending on what the committee has decided. These committees are financed by communities themselves without external support, through contributions voted on by the communities, which can be up to 9 USD per month, roughly 14% of Cameroon’s official minimum wage [35].

“*Some people say that the difference is that, self-defence is for our own safety, but Mectizan, he sees that he doesn’t gain anything in that.*”.

(Female Participant, FDG Youth, Village 1)

#### 3.3.4. Importance of Annual Training and Recycling of CDDs

Some of the community leaders that we met, especially those who were there at beginning of the CDTI, were proud because they had previously received the same training as CDDs. Beyond the simple information, the training of community leaders gives them the assurance and the responsibility to sensitize their community members. In the same vein, one community leader in our study was a pharmacist who said it was natural for him to emphasize on health in general during his mandate.

More generally, we found that the annual training and recycling of CDDs had two main advantages for the program. Firstly, training was an attracting factor for youth. During our exchanges, most of those who complained about not being approached to enlist as CDDs were frustrated because of the missed opportunity to be trained in health. Secondly, training and recycling before each ivermectin distribution campaign contributes to the appreciation of CDDs work and reassures community members. Indeed, most of the latter consider health something precious that should not be entrusted to unskilled persons, as noted by this canton leader:

“*That system of community agents recycling is good! Because you can’t go tell people that: ‘do this’, when you yourself do not know anything! How can you talk to someone about health while you do not know anything?*”

(Canton Leader, Individual Interview, Village 1)

## 4. Discussion

The main objective of the present study was to assess beneficiaries’ views on their participation in CDTI, in comparison with what was determined by WHO/APOC. We found an implementation gap in CDTI that was consistent over the 3 HDs of our study. Most of the activities under the entire communities’ charge were either undertaken by the Chief of Health Area (e.g., CDD selection) or absent (e.g., auto monitoring). These results are in line with authors’ such as Duamor et al. who recently found that only 6 of the 40 CDDs that they met were selected over a community meeting, and that the auto monitoring was not done in the surveyed communities [27]. Beyond the implementation of the CDI, one of the prerequisites for CDI sustainability is the adaptability of the approach’s principles in the local context of the beneficiaries. For instance, while the kinship organization was a contributing factor in the success of CDI implementation in Uganda [36], the caste systems and peoples’ preference to receive drugs only from health professionals were hindering factors in India [37]. 

In the present study, three main elements illustrate the fact that despite the gaps observed between the CDTI theory and practice on the field, CDI is feasible in the local context of Cameroon. Firstly, community members in general and youth in particular, were interested in the growth of their communities and were eager for information about health concerns of their communities. Secondly, even in the frame of a top-down activity, communities overwhelmingly adhered when they were convinced of the priority and accurateness of the program. Lastly, communities even contributed financially to a program when the stakes were found to be important. Besides the feasibility of CDI in the local context, we identified some factors that are important to consider in its implementation.

### 4.1. Community Participation as a Process Instead of An Intervention

As mentioned earlier, the 3 first decades of the Declaration of Alma Ata were marked by the predominance of the biomedical paradigm. Particularly in Sub-Saharan Africa, this led to the fragmentation of health systems into vertical health programs focused on diseases [3,5,8]. As highlighted by Rifkin, another result of the biomedical paradigm on community participation is that it is widely perceived as an intervention, rather than a process [3]. Consequently, focus is put on measurable and easily trackable indicators such as disease prevalence or therapeutic coverage, with less attention for community ownership, which is seen as slower and less controllable [2]. Once again, Cameroon is an example of the influence of the “trackable indicators approach”. 

At the introduction of CDTI in Cameroon, the country decided to align the strategy with the cost recovery policy that had been in place since the Initiative of Bamako [15,24]. As explained by Katabarwa et al., by 1998 several authors documented that integrating the CDTI into the cost recovery system would be important for the process’ sustainability [24]. In 2002 however, due to lower results in terms of treatment coverage, APOC and the partner NGDOs pressured the Government to exempt individuals from cost recovery fees in the context of CDTI [15,24]. The resulting decision of the Government to pay the CDDs led to irregular financial compensation [15,27]. Moreover, we recently found that the financial support of CDTI remains unclear for many Chiefs of Health Areas and CDDs, thus creating a climate of mistrust that ultimately hinders CDTI implementation at operational level [38]. 

The CDI as a process requires the involvement of many communities of different backgrounds and, for sustainability, needs to be integrated into general systems [9]. Governments and Health Planners should therefore take time and put in place comprehensive monitoring systems that will take into account indicators that are both easily quantifiable (e.g., prevalence, therapeutic coverage) and less quantifiable (example, community-ownership). For instance, the APOC experts should have considered the case of Cameroon and identified how to adapt the CDTI within the framework of the Bamako initiative. Since many Sub-Saharan African countries have signed the Bamako Initiative, lessons from this experience would have benefitted the extension of CDI to non-APOC countries that also apply the cost recovery system.

### 4.2. The Challenge of Financial Ownership of Public Health Activities

In Cameroon, the CDTI process is essentially funded by Non-Governmental Development Organizations (NGDOs) [9,27]. This contradicts the principle of beneficiaries’ ownership, as demonstrated by Rifkin et al. in 1988, readapted by Draper et al. in 2010 [29,30]. According to these authors, real community empowerment is also when community members themselves work towards finding resources, both from internal and external sources [30]. This could sound unrealistic especially for low- and middle-income countries (LMICs), but the self-defense committee that we described in our study illustrates inhabitants’ ability to choose for and finance community directed activities, even in a LMIC. In this study, socioeconomic background was a barrier for villagers, especially youth, to volunteer in CDTI. Interestingly, certain alternatives our participants mentioned during the interviews illustrated that they could discuss and adopt a consensual way of financing their CDDs if they were given such opportunity. These conclusions are nuanced however by the challenge of getting people to contribute financially for public health activities. The fact that people can finance common interest activities doesn’t necessarily mean that they will do so. After 40 years of top-down vertical public health activities provided freely, communities believe that it’s the Government’s duty to assist them and fund all the activities. Consequently, when they are asked to contribute financially in the process of community participation, they are generally reluctant, thinking that the Government is trying to escape its duties or that the local health staff are trying to divert their money [38]. These conclusions are consistent with Iyanda et al. in Nigeria and Sombié et al. in Burkina Faso, who also found that an obstacle to communities’ willingness to financially support public health activities was the perception of a Government’s disengagement [39,40]. Clear communication with communities and good governance at micro and macro political levels could therefore help to gain communities’ confidence and enhance their financial ownership of CDI. 

### 4.3. Strengthening the Operational Level Health Staff

As mentioned earlier, the local health staff usurped the decision-making roles of the communities (adopting CDTI, planning drug distribution, selecting CDDs). This similitude between 3 different HDs located in 3 different regions is an indirect sign of broader issues at stake, namely the question of real adhesion of operational level health staff to the CDTI principles.

Community participation in the CDI implies ownership and decision-making by the communities, with the local health staff playing the role of technical adviser [12]. In the literature we found evidence NGDO’s lobbied the National Health Planners [12,15,24] and communities to facilitate the implementation [15]. However, there is less evidence of such efforts towards Health District/Area staff. Yet, they are key stakeholders in the implementation of health policies and reforms, hence the term “operational level”. In a previous study conducted in the same HDs as the present, we found that Health District/Area staffs were totally dependent on upper level’s (regional or national) schedule, and either had no control over the distribution date, the number of CDDs, or the drug supply [38]. Similarly, Oyaya et al. found that while district level health staff are the ones who carry the burden of health system reforms, they are usually not involved in planning them [41].

In Cameroon, CDTI is usually introduced to operational level staff during training sessions at the beginning of annual activities, as explained by Duamor et al. [27]. In recent studies, Health District/Area staff acknowledged that despite these training sessions they didn’t give communities the opportunity to play their full role in CDTI [27,38]. Simple training is not enough to ensure Health District/Area staff’s adhesion to the principles of CDI, because most of them must shift their paradigms. In fact, operational level health staffs are generally health professionals, trained in the biomedical paradigm where there is an unequal relationship between the care provider (well informed about health and biomedical sciences) and the care seeker (often considered as “ignorant of health”). In this case, it is crucial to discuss the shift in power relations between them and their communities in the framework of CDI. Besides the power relation issues, they also experienced or heard about the previous bad experience the country had with community health workers who went beyond their mandate and acted as “little doctors” in a previous PHC activity that failed just before the introduction of CDTI [15]. As found by Meredith et al., this bad experience was one of the causes of Cameroon’s national level health planners’ reluctance towards CDTI at the beginning of the project, which they finally accepted after some lobbying by APOC and partner NGDO’s [15]. In turn, national level health planners should not only train, but also enter into dialogue with and lobby operational level health staff, with regard to the power shift on health decisions that they are supposed to support. In parallel, further studies are needed, focused on assessing those health professionals’ perceptions, fears and readiness to support total community health ownership. Findings from such studies will allow national planners to better understand the stakes, to facilitate their advocacy and to adjust the policies accordingly.

Finally, it is also important to address other issues like the real decentralization of health management and a multi sectoral approach (namely the collaboration with municipalities) in strengthening the operational level of health management.

### 4.4. Public Policies Reforms

Public policy reforms to promote and protect the health of communities are one of the 4 sets of PHC reforms that were recommended by the WHO in 2008, in the framework of the Alma Ata declaration reaffirmation [8]. In Cameroon, the fragmentation of the health system into vertical programs does not favor community participation. For instance, despite several national guidelines and the presence of a ministerial department in charge of health promotion, Njepel et al. found many weaknesses in the setting out of health promotion namely as regards the Ottawa Charter [42]. These authors concluded that in the Sectoral Strategy for Health (SSH), the reference document for health interventions for the period 2001–2015, most of the activities were focused on the individual level of the Charter, using only health education on priority health programs [42]. In the latest SSH document released at the end of 2016, community participation is embedded in the CDI [43] and other strategies such as multisectoral collaboration and decentralization are evoked for better results in people’s health in general. However, these strategies were also mentioned in earlier versions of the SSH and have been poorly implemented [42]. Further health policy studies would provide better insights into the application of the new SSH, and its effects on the health system in general, as well as into CDI process implementation. 

Finally, international partners also have their role to play in order to facilitate the effective comprehensiveness of CDI. The real challenge here is donors’ readiness to invest together in a complex process, of which the impact (community participation) is neither clearly nor easily measurable [2,3]. In such a complex system, it is far from obvious to link the results to a specific action. This could explain the difficulties for donors to invest in a “common basket”, given the context of persisting “rivalries between diseases” in the global health community [44,45]. 

### 4.5. Study Limitations

This study showed the feasibility of CDI in rural and semi-urban zones of Cameroon. Our key findings are consistent with those of other authors who worked in similar settlements in the country, which indicates their reliability. However, cautions should be taken when applying these results to urban contexts, particularly as regards communities’ perspectives. In fact, urbanization, better access to New Information and Communication Technologies, diversity of cultures and frequent population migration are examples of factors that lead to different socio cultural or epidemiological features in urban settlements. The particularities of the urban context in Africa are also noted by other authors such as Ajayi et al. in Nigeria and Odhiambo et al. in Kenya [46,47]. In order to extend the CDI to the whole country, epidemiological, entomological and socio-anthropological studies are needed to enrich the knowledge about the urban specificities, both in urban rich and urban poor settlements.

Another limitation could reside in the fact that qualitative research results cannot be generalized, mainly due to the number of participants. However, there are several criteria that are used to minimize this limitation and enhance the quality and external validity of qualitative researches. These include credibility (respondents triangulation, investigators triangulation, member checking) and transferability (resonance with existing literature) [48,49]. As it pertains to triangulation, study respondents came from three different regions of the country, and in each health district we recruited from two different Health Areas. We also ensured variability in age, experience and gender in the choice of our participants. Furthermore, we applied the principle of interrater reliability, in which raw data were independently reviewed by different authors. The results were afterwards compared and discussed together.

## 5. Conclusions

Four decades after the Declaration of Alma Ata, community ownership in CDI process is still low in Cameroon. The concept of CDI is culturally feasible in rural and semi-urban settlements, but many challenges hinder its real implementation. In the view of community participation as a process rather than an intervention, these challenges include real dialogue with communities as partners, dialogue and advocacy with operational level health staff, and macro-economic and political reforms in health, finance and other associated sectors.

## Figures and Tables

**Table 1:** Process and role distribution in the CDTI/CDI ^1^.

	Activities	Actors Responsible of the Activities	Interlocutors/Beneficiaries
PRE-COMMUNITY PHASE	Advocacy	Partners, NGDOs, National Level Health Planners	National, sub-national and district planners (in health and other partners from public/private sectors)
	Generate health policies and guidelines for the CDI intervention package	National Level Health Planners	Regional, District and Health area officials
	Training of the District and Health Area Staff	National/Regional health staff	District and Health area officials
COMMUNITY PHASE	Introduce to the head of a community	Health Area Official	Community leader
	Explain the CDTI principles to the community	Health Area Official	Entire community
	Decision to adopt the CDTI strategy; Planning of period and modalities of ivermectin distribution (how and where); Election of CDDs and decision of their incentive’s modalities	Entire community	Entire community
	Feed Back to the Health Area Officials	Entire community	Health Area Official
	Training of CDDs	Health Area Official	Selected CDDs
	Census of the community and estimation of ivermectin doses needed	Selected CDDs	Entire community
	Collection of ivermectin from the Health Area Officials and distribution to the community	Selected CDDs	Entire community
	Monitoring of the community distribution process	Health Area Official	Selected CDDs
	Community Auto monitoring of the results of the intervention	Entire community	Entire community
	Report of distribution results to the Health System	Entire community	Health Area Official

^1^ Adapted from WHO/APOC and The CDI Study Group [12,32]. Abbreviations: CDDs community directed distributors; CDI community directed interventions; CDTI community directed treatment with ivermectin; NGDOs non-governmental development organizations.

**Table 2:** Characteristics of the participants of individual interviews.

Participants	Role	Gender	Qualification/Profession
Participant 1	community leader	Male	community leader
Participant 2	community leader	Male	community leader
Participant 3	canton leader	Male	canton leader
Participant 4	community leader	Male	farmer
Participant 5	community leader	Male	farmer
Participant 6	community leader	Male	trader
Participant 7	community leader	Male	self employed
Participant 8	CDTI averse	Male	farmer/trader
Participant 9	CDTI averse	Male	farmer/trader
Participant 10	CDTI averse	Female	trader
Participant 11	CDTI averse	Female	farmer

**Table 3:** Characteristics of the participants in Focus Group Discussions (FGD).

	Number of Participants	Gender Distribution	Age Description of the Group
FGD1	7	7F	elders (≥35 years)
FGD2	5	2F/3M	youth (16–25 years)
FGD3	9	5F/4M	elders (≥35 years)
FGD4	8	5F/3M	youth (16–25 years)
FGD5	6	2F/4M	youth (16–25 years)
FGD6	6	3F/3M	elders (≥35 years)
FGD7	8	5F/3M	elders (≥35 years)
FGD8	8	2F/6M	youth (16–25 years)
FGD9	6	6M	elders (≥35 years)
FGD10	14	8F/6M	youth (16–25 years)

Abbreviations: F female participant; FGD focus group discussion; M male participant.

**Table 4:** Comparison of roles distribution in the CDTI between WHO/APOC theory and communities’ perceptions.

Activities	Actors Responsible of the Activities According to WHO/APOC Theory	Actors Conducting the Activities According to Our Participants’ Views
Introduce to the head of a community	Health Area Official	Health Area Official
Explain the CDTI principles to the community	Health Area Official, during a general assembly	**Not Done** ^1^
Decision to adopt the CDTI strategy; Planning of period and modalities of ivermectin distribution (how and where); Nomination of CDDs and decision of their incentive’s modalities	Entire community	**Health Area Official**
Feed Back to the Health Area Officials	Entire community	**Not Done**
Training of CDDs	Health Area Official	Health Area Official
Census of the community and estimation of ivermectin doses needed	Selected CDDs	Selected CDDs
Collection of ivermectin from the Health Area Officials and distribution to the community	Selected CDDs	Selected CDDs
Monitoring of the community distribution process	Health Area Official	Not discussed with the participants
Community Auto monitoring of the results of the intervention	Entire community	**Not Done**
Report of distribution results to the Health System	Entire community	**Selected CDDs**

^1^ In bold: Discordance with theory. Abbreviations: APOC African program for onchocerciasis control; CDDs community directed distributors; CDTI community directed treatment with ivermectin; WHO world health organization.

## Supplementary Materials

The following are available online at https://www.mdpi.com/2414-6366/4/3/105/s1, Interviews and FGDs original guides (in French).

## Abbreviations

AIDS	acquired immunodeficiency syndrome
APOC	African program for onchocerciasis control
CBIT	community based ivermectin treatment
CDDs	community directed distributors
CDI	community directed interventions
CDTI	community directed treatment with ivermectin
CHAs	chiefs of health area
CP	community participation
FGDs	focus group discussions
HD	health district
HIV	human immunodeficiency virus
LMICs	low and middle income countries
NGDOs	non-governmental development organizations
PHC	primary health care
SSH	sectoral strategy for health
WHO	world health organization
