# Supplementary material for: Do Communities Really “Direct” in Community-Directed Interventions? A Qualitative Assessment of Beneficiaries’ Perceptions at 20 Years of Community Directed Treatment with Ivermectin in Cameroon
Source: Trop Med Infect Dis. 2019 Jul 15;4(3):105. doi: 10.3390/tropicalmed4030105 (PMC6789878; doi:10.3390/tropicalmed4030105)
Supplement: Supplementary file 1 [file tropicalmed-04-00105-s001.pdf]

PROJET DE RENFORCEMENT DU PROGRAMME D'ELIMINATION DE L'ONCHOCERCOSE AU CAMEROUN (PRD 2013 CMR SOUOPGUI)

GUIDES D'ENTRETIENS INDIVIDUELS ET FOCUS GROUP DISCUSSIONS

1. Présentation de la recherche

- Nom et rôle du chercheur
- Cadre et but de la recherche
- Procédure de collecte des données : échanges enregistrés si participant(s) d'accord. Raisons pour lesquelles le chercheur voudrait avoir des enregistrements des échanges.
- Grandes lignes des échanges
- Questions éthiques : confidentialité et utilisation des données (conservation, partage)

2. Grilles d'entretiens semi-directifs pour personnes clés

2.1 Entretiens avec les chefs de villages

THEMATIQUE	QUESTION PRINCIPALE	QUESTIONS DE RELANCE POSSIBLES	INTERET (S) DE LA QUESTION
Organisation générale du village/communauté	**Parle-moi de ton village et de toi en tant que chef de village ?**	- Combien de personnes dans le village ?<br>- Qui sont les plus nombreux entre les hommes et les femmes / entre les jeunes et les vieux ?<br>- Depuis quand est chef ?<br>- Comment devient-on chef ?<br>- Quelles sont les tâches/responsabilités d'un chef ?	1. Brise-glace<br><br>2. Introduire sur l'organisation habituelle de la communauté
Lutte contre la maladie	**Comment est-ce que les gens de votre village se soignent quand ils sont malades ?**	- Quelles sont les principales maladies du village ?<br>- Comment les villageois font-ils pour lutter contre ces maladies-là ?	Comprendre l'organisation de la communauté face aux maladies en général

		- Quelle est la place du chef du village dans la lutte contre les maladies ?	
Lutte contre la maladie	**Quels sont les modes de fonctionnement du Comité de Santé de ce village ?**	- Comment fait-on pour devenir membre de ce comité ?<br>- Comment le président est-il nommé ?<br>- Y a-t-il des déserteurs ? Des volontaires récusés ? Raconter des exemples<br>- Que fait le COSA concrètement dans la lutte contre les maladies citées ?	Comprendre l'organisation de la communauté face aux maladies en général
Lutte contre l'onchocercose (déroulement des campagnes)	**Parlant de l'onchocercose/filaires, raconte-moi comment dans ce village vous faites pour combattre cette maladie ?**	-y a –t-il des campagnes :<br>*D'information et sensibilisation sur les filaires ?<br>*D'organisation de distribution de médicaments<br>*d'assainissement de l'environnement<br>Qu'est ce qui se passe pendant les campagnes ?<br>- Comment se fait la sélection des DC ?<br>- Qu'est-ce que vous pensez des DC ?<br>- Comment le reste de la communauté est-elle ou pas impliquée ?<br>- Comment se passe la lutte entre les campagnes ?<br>- Qui sont ceux qui ne s'impliquent pas dans la campagne ni ne prennent le Mectizan et pourquoi ?	1. Décrire l'organisation de la communauté face à l'onchocercose<br>2. Avoir des pistes quant au profil type des réfractaires
Participation communautaire	**Comment peux-tu décrire le rôle qu'on te donne en tant que chef**	- Que fait concrètement le chef de village pendant les campagnes ?	1. Comprendre la perception des acteurs clés sur la participation

	**dans les activités d'onchocercose ?**	- Comment les dates des campagnes sont-elles fixées ?<br>- Quelles sont les activités menées juste après la campagne ?<br>- Que fait concrètement le chef de village en dehors des campagnes ?<br>- Raconte-moi, quelles sont les choses que tu voudrais faire en tant que chef dans la lutte contre l'oncho/filaires mais que tu ne fais pas ?<br>- A l'inverse, décris-moi certaines activités qu'on te demande de faire et qui à ton avis ne relèvent pas de tes fonctions de chef ?	communautaire dans le cadre du système TIDC<br><br>2. Rechercher la mise en œuvre des activités d'auto-monitorage<br><br>3. Comprendre les attentes en termes de participation aux activités
	**Comment tu décris tes relations avec les autres acteurs ?**	- Relations avec le chef de l'aire<br>- Relations avec le président du comité de santé<br>- Relations avec le chef de District	
Clôture	*Y a-t-il autre chose qui est importante et que tu veux soulever ici ?*	*- est ce que tu as des questions ?*<br>*- est ce que tu as des commentaires à faire par rapport à ce dont on a discuté ?*	*Clôture*<br>*Laisser la possibilité au participant de revenir sur ce qui a été discuté : ajouts, rectificatifs.*

2.2 Entretiens avec les réfractaires au TIDC/Campagnes de distribution de l'ivermectine

THEMATIQUE	QUESTION PRINCIPALE	QUESTIONS DE RELANCE POSSIBLES	INTERET (S) DE LA QUESTION
Organisation générale de la famille	**Parle-moi un peu de toi et ta famille**	- Depuis quand est tu dans le village ?<br>- De quelle ethnie ?<br>- quel est ton travail,<br>- Combien êtes-vous dans ta famille ?	Brise-glace
Lutte contre la maladie	**Comment est-ce que toi et ta famille vous vous soignez quand vous êtes malades ?**	- Quelles sont les principales maladies auxquelles vous faites face ?	Comprendre l'organisation de la communauté face aux maladies en général et dans le

		- Comment faites-vous pour combattre ces maladies ?<br>- Comment les autres villageois font-ils pour lutter contre ces maladies-là ?<br>- est ce que toi ou ta famille organisez souvent des séances d'assainissement de l'environnement ou autres actions du genre pour prévenir les maladies ?	cadre de la prévention de la maladie.
Lutte contre la maladie	**Y a-t-il un comité de santé dans ce village ?<br>- Si oui, quels sont les modes de fonctionnement du Comité de Santé de ce village ?**	- Comment fait-on pour devenir membre de ce comité ?<br>- Comment le président est-il nommé ?<br>- Y a-t-il des déserteurs ? Des volontaires récusés ? Raconter des exemples<br>- Que fait le COSA concrètement dans la lutte contre les maladies citées ?	Comprendre l'organisation de la communauté face aux maladies en général
Lutte contre l'onchocercose	**Parlant de l'onchocercose/filaires, raconte-moi comment dans ta famille on fait pour combattre cette maladie ?**	- Est-ce que toi ou un proche a déjà souffert de l'onchocercose, si oui raconter comment cela s'est passé.<br>- Peux-tu me raconter comment ça se passe quand vous prenez le Mectizan ?<br>(<u>Si personne réfractaire</u> :<br>- Préciser si jamais pris ou abandon ? expliquer les motivations de non prise/abandon du traitement.<br>- Si pas encore abordé, questionner sur les alternatives utilisées)	1. Décrire l'organisation de la communauté face à l'onchocercose<br><br>2. Comprendre les motivations des personnes réfractaires

		- En dehors des campagnes, qu'est-ce qui est fait dans le village pour combattre l'onchocercose ?	
Participation communautaire	**Comment peux-tu décrire la place qu'on te donne en tant qu'habitant dans les activités d'onchocercose ?**	y a –t-il des campagnes :<br>*D'information et sensibilisation sur les filaires ?<br>*D'organisation de distribution de médicaments<br>*d'assainissement de l'environnement<br>Qu'est ce qui se passe pendant les campagnes ?<br>- Raconte-moi, quelles sont les choses que tu fais en tant que habitant de ce village?<br>- Raconte-moi, quelles sont les choses que tu voudrais faire en tant que habitant de ce village mais que tu ne fais pas dans la lutte contre l'oncho ? (si répond 'rien', le relancer sur les raisons de démotivation)<br>- Raconte-moi quelles sont les choses que tu fais, mais tu penses que ce n'est pas aux habitants de les faire ? (Pourquoi ces activités ne sont pas réservées aux habitants et à qui sont-elles réservées ?)<br>- Quelles sont les activités menées juste après la campagne ?	1. Comprendre la perception des acteurs clés sur la participation communautaire dans le cadre du système TIDC<br><br>2. Comprendre les attentes en termes de participation aux activités<br><br>3. Rechercher la mise en œuvre des activités d'auto monitorage
Clôture	*Y a-t-il autre chose qui est importante et que tu veux soulever ici ?*	*- est ce que tu as des questions ?*<br>*- est ce que tu as des commentaires à faire*	*Clôture*<br>*Laisser la possibilité au participant de revenir sur ce qui a*

		par rapport à ce dont on a discuté ?	*été discuté : ajouts, rectificatifs.*

3. <u>Focus group discussions avec les habitants du village</u>

THEMES A DEBATTRE	SOUS THEMES POSSIBLES	JUSTIFICATION
Présentation des participants	Chacun donne sa profession et son lieu de résidence	Brise-glace
**Comment se passent les campagnes de distribution de Mectizan dans votre quartier**	- la ou les maladies ciblées<br>- durée des campagnes<br>- quels sont ceux qui distribuent<br>- qui sont ceux qui en prennent dans la communauté ?<br>- qui sont ceux qui n'en prennent pas et pourquoi<br>- quels sont les points forts<br>- quels sont les points faibles<br>- est ce que les gens de mon entourage aiment ces campagnes ?<br>- quelle est l'expérience de ceux qui prennent le Mectizan	Avoir une idée de l'organisation locale telle que vécue par les individus
**Participation communautaire : est ce que vous participez/êtes impliqués dans les activités de santé de votre village ?**	- nécessité ou pas de participer en tant que population, avec exemples de participation<br>- qu'est-ce que les habitants de quartier font actuellement ?<br>- est-ce qu'il est possible de faire mieux ?<br>- qu'est ce qui n'est pas possible ou ne devrait pas être demandé aux populations ?<br>- à part l'onchocercose quels autres programmes où il est demandé de participer ?<br>- à part l'onchocercose, quels autres programmes vous auriez souhaité participer et de quelle manière.	1. Comprendre la perception des acteurs clés sur la participation communautaire dans le cadre du système TIDC<br><br>2. Comprendre les attentes en termes de participation aux activités
Autre chose importante à mentionner ?	*- est ce que quelqu'un a des questions ?*<br>*- est ce que quelqu'un a des commentaires à faire par rapport à ce dont on a discuté ?*	*Clôture*<br>*Laisser la possibilité aux participants de revenir sur ce qui a été discuté : ajouts, rectificatifs.*
